# Novel Patterns of Photosynthetic Activity Within Canopies of Poplar Stands with Different Densities

**DOI:** 10.3390/plants14060898

**Published:** 2025-03-13

**Authors:** Taijin Zhang, Xiaoting Liu, Qinhui Zhang, Hui Xiao, Yunong Han, Zhenghua Xing, Fusen Wang, Xiyang Zhao

**Affiliations:** 1 Jilin Provincial Key Laboratory of Tree and Grass Genetics and Breeding, College of Forestry and Grassland Science, Jilin Agricultural University, Changchun 130118, China; zhangtaijin0210@mails.jlau.edu.cn (T.Z.);; 2 National Key Laboratory of Forest Genetics and Breeding, Northeast Forestry University, Harbin 150040, China; 3 Qiqihar Branch of Heilongjiang Academy of Forestry, Qiqihar City 161000, China

**Keywords:** canopy layer, leaf traits, photosynthetic traits, planting density, *Populus euramericana* ‘N3016’ × *Populus ussuriensis*

## Abstract

(1) Background: Planting density is an important factor affecting the yield of poplar per unit area. Therefore, determining the optimal height of the photosynthetic canopy layer for different planting densities is critical. (2) Methods: This study takes *Populus euramericana* ‘N3016’ × *Populus ussuriensis* as the research object. According to on the average tree height, diameter at breast height, and crown width of the stand, one standard tree was selected from each planting density for the experiment. The canopy of the standard tree was divided into five canopy layers from top to bottom, and the first-order lateral branches of each canopy layer were divided into three sites from outside to inside. The photosynthesis and leaf traits at various positions in different canopy layers were measured. (3) Results: The results revealed significant differences in photosynthetic and leaf traits at different positions of different canopy layers under different planting densities. As the canopy layer gradually declined, photosynthetic traits revealed that instantaneous photosynthetic rate (Pn), transpiration rate (Tr), and stomatal conductance (Gs) gradually decreased, while intercellular CO_2_ concentration (Ci) increased. Moreover, water use efficiency (WUE) initially increased and then decreased under an 825 trees·ha^−1^ (D3) planting density. Leaf traits revealed that as leaf length (LL) gradually decreased, leaf width (LW), leaf area (LA), and leaf water content (LWC) gradually increased. Under three planting densities, leaf traits were negatively correlated with Pn, Tr, WUE, and Gs, but positively correlated with Ci. (4) Conclusions: As the planting density decreased, the photosynthetic capacity of poplar gradually increased. With a planting density of D3, all canopy layers were able to carry out efficient photosynthesis, and all living branches within the canopy were functional. However, under the planting density of 1650 trees·ha^−1^ (D1) and 1089 trees·ha^−1^ (D2), canopy layers 1 to 4 could perform effective photosynthesis, while the photosynthetic capacity of canopy layer 5 was relatively weak. This study reveals the interactive effects of canopy position and stand density on leaf physiological and morphological traits, providing new insights into the photosynthetic efficiency and growth strategies of poplar under different planting densities. It also offers theoretical support for optimizing stand management and enhancing productivity.

## 1. Introduction

Photosynthesis is a crucial process for plant growth as it provides the necessary material and energy for plant development, forming the basis of plant yield [1,2]. It is a crucial biochemical process in plant growth and development, with its efficiency regulated by a combination of internal and external factors. In addition to the plant’s inherent physiological traits, changes in canopy density not only significantly alter the light distribution within the stand but also indirectly modify environmental factors such as carbon dioxide concentration, water availability, and temperature, thereby exerting multi-layered effects on photosynthesis [3,4,5]. In this process, the plasticity response of leaf phenotypes is particularly important, as the morphology and structural composition of leaves directly affect photosynthetic efficiency. Studies have shown that leaf morphology undergoes significant changes in response to variations in factors such as light intensity, temperature, and moisture conditions [6]. Under low-light conditions, the leaf area typically increases to enhance light capture efficiency, whereas excessively high light intensity may lead to leaf dormancy to reduce photodamage [7]. Additionally, the structure of the tree canopy is crucial for plant photosynthesis, and different planting densities directly influence the light intensity within the canopy, thereby altering leaf morphology and photosynthetic efficiency [8].

During photosynthesis in trees, the net photosynthetic yield of branches in each canopy layer is the main source of trunk growth. However, not all live branches contribute to the growth of the trunk, as lower canopy branches often have weak photosynthetic capacity due to shading from adjacent trees and upper layers [9,10], sometimes even drawing nutrients from the trunk [11]. Since the advent and application of the infrared gas analyzer (IRGA) in plant photosynthesis research, studies on photosynthetic gradients in trees have gradually increased. The earliest research can be traced back to the late 1940s and early 1950s, primarily conducted in Austria, Czechoslovakia, and Germany. Studies have shown that the photosynthetic rate in the upper canopy of trees is significantly higher than that in the lower canopy. High planting density can alter light distribution and canopy structure, potentially leading to a decline in the photosynthetic capacity of lower leaves, thereby affecting the overall photosynthetic efficiency and productivity of the trees [12,13,14,15]. Moreover, under different planting densities, the photosynthetic capacity of trees varies. Stands with higher planting density have an uneven distribution of light, water, and carbon dioxide between the canopy layers. Therefore, the upper leaves of trees receive more light, while the lower leaves receive less light, affecting the photosynthetic efficiency of trees [16,17,18]. Additionally, studies have shown that improper distribution of photosynthesis across different growth stages can also lead to a reduction in light energy utilization efficiency, directly impacting stand productivity [19,20]. Consequently, determining the effective canopy layer height of trees within a suitable planting density plays an important role in the growth and development of trees. Properly regulating planting density and canopy structure can not only optimize the distribution of light resources but also enhance the overall photosynthetic efficiency and trunk growth rate of trees.

Poplar is a general term for a class of deciduous trees of the genus *Populus* (Salicaceae), which is mostly distributed in the temperate regions of the northern hemisphere. It exhibits the characteristics of rapid growth, early maturity, strong adaptability, widely used wood, and easy renewal. Poplar is one of the most widely planted tree species domestically and abroad, and it is also one of the fastest-growing timber species with the largest cultivated area and the highest timber yield in the middle latitude plains of the world. It can be used as a protection forest, timber forest, garden application, for papermaking, and various other uses. It is an important, rapidly growing, and high-yielding forest tree species in China. *Populus euramericana* ‘N3016’ × *Populus ussuriensis* has excellent characteristics such as a straight trunk, fast growth, excellent material quality, cold resistance, drought resistance, and pest resistance [21]. It is suitable for planting in high latitudes and extremely cold areas due to its strong adaptability. It is a favorable choice for planting tree species in low temperatures and poor soil areas in Northeast China. Simultaneously, *P. euramericana* ‘N3016’ × *P. ussuriensis* is suitable for industrial timber such as pulpwood, protective forests, and landscaping.

Numerous studies have been conducted on the Pn of different canopy layers of poplar. However, there are few studies on the effective Pn of each canopy layer of poplar under different planting densities. The planting density is an important factor affecting the yield of poplar unit area and determining the effective photosynthetic crown width under different planting densities. This study aimed to determine the photosynthetic traits and leaf traits of different lateral branch positions of each canopy layer of poplar under different planting densities. Additionally, this study analyzed how changes in stand density affected positional differences in photosynthetic and leaf morphological traits to clarify the effective canopy layer height of poplar in a suitable planting density. It was also designed to enhance the growth rate of poplar and the yield and quality of afforestation, offering a scientific basis for the efficient breeding of poplar in Northeast China.

## 2. Results

### 2.1. ANOVA of Photosynthetic Traits and Leaf Traits

The photosynthetic and leaf traits of various canopy layers within different positions under different planting densities were analyzed using ANOVA (Table 1). The results revealed significant differences in photosynthetic traits under various planting densities, different canopy layers, and different positions (*p* < 0.01). There were also significant differences in photosynthetic traits under the interaction of planting density and canopy layer, planting density and position, canopy layer and position, and all three factors together (*p* < 0.01). Leaf traits were significantly different under different planting densities and different canopy layers (*p* < 0.01), and the leaf area (LA) was significantly different under different positions (*p* < 0.01). Additionally, leaf length (LL), leaf width (LW), and LA were significantly different under the interaction of planting density and canopy layer, and canopy layer and position (*p* < 0.01). LW was also significantly different under the interaction of planting density and position (*p* < 0.05). LL was significantly different under the interaction of planting density, canopy layer, and position (*p* < 0.05), while LW and LA were significantly different (*p* < 0.01).

### 2.2. Mean Value Analysis of Photosynthetic Traits at Different Planting Densities, Different Canopy Layers, and Different Positions

**Figure 1:**
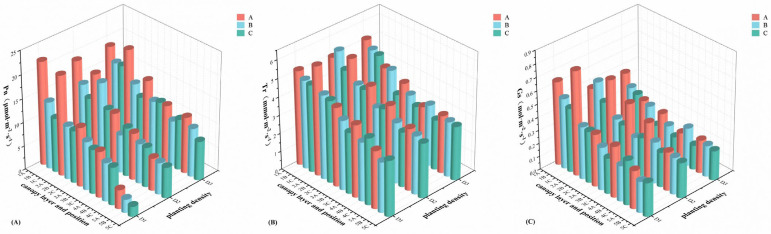
Three-dimensional analysis of instantaneous photosynthetic rate (Pn) (**A**), transpiration rate (Tr) (**B**) and stomatal conductance (Gs) (**C**) at different planting densities, different canopy layers and different positions. A, B, and C represent the position of the lateral branch, from the outside to the inside, in turn, as position A, position B, and position C; abscissas 1, 2, 3, 4, and 5 represent canopy layer 1, canopy layer 2, canopy layer 3, canopy layer 4, and canopy layer 5, respectively; D1, approximately 1650 trees·ha^−1^; D2, approximately 1089 trees·ha^−1^; D3, approximately 825 trees·ha^−1^.

The mean value analysis of instantaneous photosynthetic rate (Pn), transpiration rate (Tr), and stomatal conductance (Gs) at different positions of different canopy layers under different planting densities is displayed in Figure 1. The results reveal that all Pn, Tr, and Gs of different canopy layers of different planting densities gradually decreased from top to bottom, with effective photosynthesis observed from canopy layer 1 to 4. Their maximum values all occurred in canopy layers 1 and 2. Under the same canopy layer conditions, Gs did not show a significant pattern across different planting densities, and Pn followed the order D3 > D2 > D1 from canopy layer 3 to 5, while Tr followed the order D2 > D1 > D3. Additionally, all Pn, Tr, and Gs exhibited a decreasing trend from the outer to the inner positions within the same canopy layer across all planting densities. Notably, under the D3 planting density, the Pn of canopy layer 5 was similar to that of canopy layer 4 under the other two planting densities, ranging from 8 to 11 μmol·m^−2^·s^−1^, indicating that effective photosynthesis can be sustained in all canopy layers at the D3 planting density.

**Figure 2:**
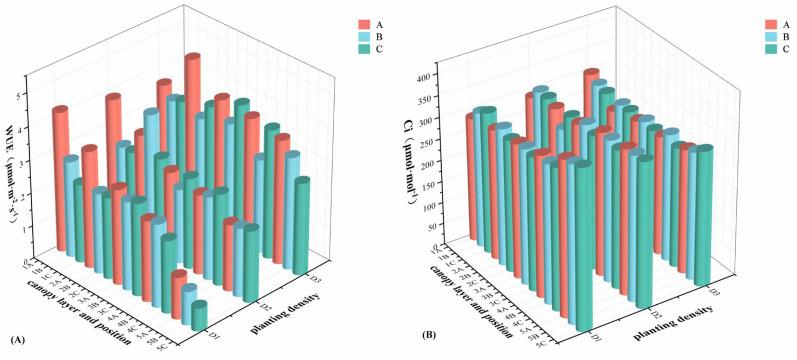
Three-dimensional analysis of water use efficiency (WUE) (**A**), intercellular CO_2_ concentration (Ci) (**B**) at different planting densities, different canopy layers, and different positions. A, B, and C, represent the position of the lateral branch from the outside to the inside, in turn, as position A, position B, and position C; abscissas 1, 2, 3, 4, and 5 represent canopy layer 1, canopy layer 2, canopy layer 3, canopy layer 4, and canopy layer 5, respectively; D1, approximately 1650 trees·ha^−1^; D2, approximately 1089 trees·ha^−1^; D3, approximately 825 trees·ha^−1^.

The mean value analysis of water use efficiency (WUE) and intercellular CO_2_ concentration (Ci) at different positions of different canopy layers under different planting densities is displayed in Figure 2. The results of Ci revealed that Ci gradually increased from the top to the bottom canopy layers under different planting densities. In the same canopy layer, Ci at the D3 planting density was significantly lower than that at the D1 and D2 planting densities. Ci at different positions within the same canopy layer exhibited a gradually increasing trend from the outside to the inside across all planting densities. The results of WUE revealed that WUE gradually decreased from the top to the bottom canopy layers under the D1 and D2 planting densities. However, under the D3 planting density, WUE exhibited a trend of initially increasing and then declining from the top to the bottom canopy layers. The WUE of D3 was significantly higher than that of the other two planting densities, with values ranging from 2.79 to 4.80 μmol·m^−2^·s^−1^. The WUE of the same canopy layer with different planting densities was D3 > D2 > D1. Moreover, within the same canopy layer, WUE under the D1 planting density decreased gradually from the outside to the inside, while under the D2 and D3 planting densities, it initially decreased and then increased.

### 2.3. Analysis of Leaf Traits at Different Planting Densities, Different Canopy Layers, and Different Positions

**Figure 3:**
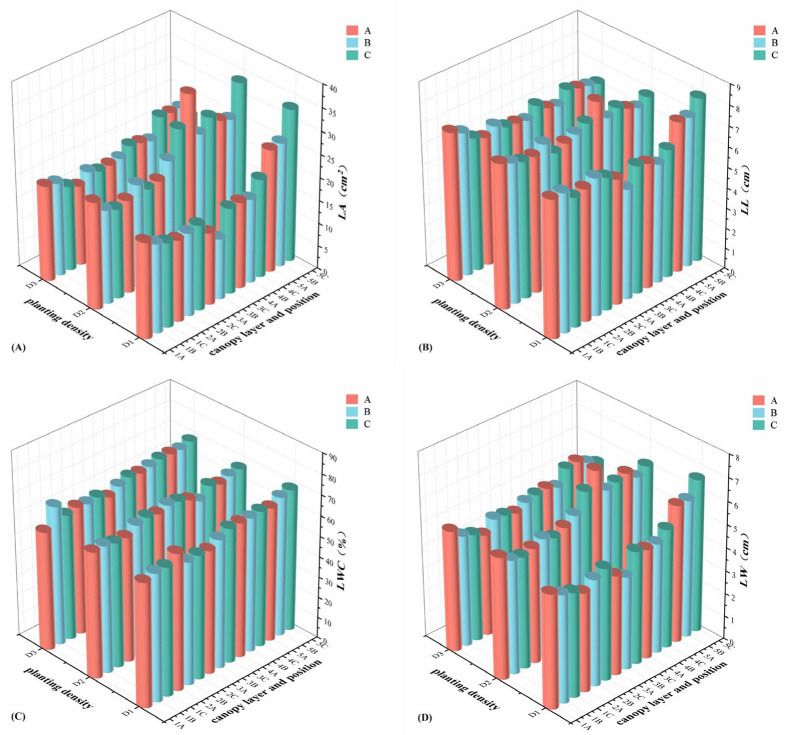
Three-dimensional analysis of leaf area (LA) (**A**), leaf length (LL) (**B**), leaf water content (LWC) (**C**), and leaf width (LW) (**D**) at different planting densities, different canopy layers, and different positions. A, B, and C represent the position of the lateral branch, from the outside to the inside, in turn, as position A, position B, and position C; the abscissas 1, 2, 3, 4, and 5 represent canopy layer 1, canopy layer 2, canopy layer 3, canopy layer 4, and canopy layer 5, respectively; D1, approximately 1650 trees·ha^−1^; D2, approximately 1089 trees·ha^−1^; D3, approximately 825 trees·ha^−1^.

The mean value analysis of LL, LW, LA, and LWC at different positions within various canopy layers under different planting densities is displayed in Figure 3. Under different planting densities, LL exhibited a trend of initially decreasing and then increasing from the upper to the lower canopy layers, while LW, LA, and LWC showed a gradual increase. In canopy layer 1, no clear patterns were observed for leaf traits across different planting densities. However, in canopy layers 2 to 4, the maximum values of LL, LW, and LA were consistently observed under the D2 planting density, following the order D2 > D3 > D1. Notably, in canopy layer 5, LL, LW, and LA under the D1 planting density were significantly higher than those under D2 and D3, with the order D1 > D2 > D3. Within the same canopy layer under different planting densities, LA exhibited a gradually increasing trend from the outer to the inner positions, while no clear patterns were observed for other leaf traits. Additionally, LWC did not show significant patterns across different planting densities, canopy layers, or positions.

### 2.4. Correlation Analysis of Photosynthetic Traits and Leaf Traits

The study analyzed the correlation between photosynthetic and leaf traits at different positions of each canopy layer under different planting densities. The results are detailed in Table 2. There was a highly significant positive correlation between Pn and Tr, WUE, and Gs (0.586 < *r* < 0.784, *p* < 0.01). Tr displayed a highly significant positive correlation with WUE and Gs (0.154 < *r* < 0.906, *p* < 0.01). Among leaf traits, LL displayed a significant positive correlation with LW and LA (0.400 < *r* < 0.671, *p* < 0.01). LW also exhibited a highly significant positive correlation with LA and LWC (0.444 < *r* < 0.471, *p* < 0.01). Moreover, there was a highly significant positive correlation between LA and LWC (*r* = 0.368, *p* < 0.01). Between photosynthetic and leaf traits, Ci exhibited a highly significant positive correlation with LL, LW, and LA (0.195 < *r* < 0.545, *p* < 0.01).

## 3. Discussion

Photosynthesis is a crucial physiological process for plants as it forms the basis of energy flow and material circulation in forest ecosystems, providing plants with the energy needed for growth and metabolism [22]. There are significant differences in photosynthesis due to environmental factors such as light intensity, temperature, moisture, and CO_2_ concentration. In stands with different planting densities, the light intensity varies among canopy layers, leading to changes in temperature, water, and other factors, ultimately resulting in significant differences in Pn [15,23]. Studies have revealed that the plant Pn changes with the change in light intensity. Optimal light conditions can enhance the growth of plants, while excessive light can lead to photoinhibition [24].

In photosynthesis, the Pn can directly reflect the efficiency of photosynthesis, indicating plant growth and productivity [25]. A greater Pn indicates that the plant contains more nutrients [26]. In this study, Pn of the three planting densities exhibited a decreasing trend with the decline in the canopy layer. However, the Pn decreased with the increase in planting density from canopy layer 3 to 5. This may be caused by the decrease in light transmittance and the mutual occlusion between tree branches under high planting density, leading to a significant decrease in light intensity [27]. In addition, at varying planting densities, the Pn exhibited a decreasing trend from outside to the inside at different positions within each canopy layer. This could be due to the trunk requiring lateral branches to provide nutrients for its own growth. However, the growth direction of the lateral branches from the trunk is oblique and upward. Therefore, the lateral branches near the trunk need to provide nutrients for their own growth and development as well as for the trunk, leading to a lower Pn for the lateral branches near the trunk compared to the other lateral branches [28].

In this study, the Pn of canopy layer 5 under the planting density of 4 × 3 × 6 m was similar to that of the canopy layer 4 under the other two planting densities. After pruning, the percentage of diameter growth at breast height of the planting density was negative. This may be due to the low stand crown density under this planting density, allowing each canopy layer to carry out effective photosynthesis [29]. The branches of canopy layer 5 can effectively provide nutrients for the growth of the trunk and significantly contribute to poplar growth. However, pruning removes this effective canopy layer, resulting in a slower growth rate of the stand at this planting density. Conversely, in the other two planting densities, the percentage of diameter at breast height growth after pruning is above 10%, indicating that the lateral branches of canopy layer 5 at this planting density cannot carry out effective photosynthesis and cannot provide nutrients for tree growth. Inversely, they consume the nutrients of the trunk, leading to an increase in the annual growth of diameter at breast height after pruning [11,30].

Gs is an indicator to measure the degree of stomatal opening of plants. Stomata are the main channels for gas exchange between plant leaves and the outside environment [31]. The degree of stomatal opening is affected by light, temperature, and humidity, while the stomatal size leads to a change in gas exchange, water utilization, and transpiration [32]. In this study, with the gradual increase in the canopy layer, Gs also gradually increased under three planting densities. This is because as the canopy layer gradually increased, the light intensity and temperature increased, requiring the plant to increase Gs to accelerate Tr to reduce high-temperature stress [33]. However, excessive temperature may also lead to stomatal closure [34].

Tr refers to the amount of water transpired per unit LA of plants within a specific period. Factors such as photosynthetically active radiation, temperature, and humidity will affect the Tr of plants [35]. Simultaneously, plants regulate the Tr by adjusting the stomatal state of their leaves to adapt to environmental changes and maintain water balance. In this study, with the increase in planting density, the Tr exhibited an initial increasing trend and then decreased from canopy layer 3 to 5. This could be attributed to the moderate crown density between trees under the D2 planting density, where light was no longer the only limiting factor. Poplar achieved maximum photosynthetic benefits by regulating nutrient balance and increasing the Tr [18]. Additionally, under different planting densities, the Tr decreased significantly as the canopy layer gradually declined. This decline of the canopy layer can be attributed to weakened light intensity received by each canopy layer, increased canopy layer density, increased relative humidity of the air, and decreased temperature, leading to a significant decrease in the Tr [36]. This is also the reason why the Tr at different positions gradually decreases from the outside to the inside.

WUE reflects the relationship between plant water consumption and dry matter yield. It is an index used to evaluate the adaptability of plant growth to the environment [37,38]. The change in WUE is directly related to the Pn and Tr. In this study, with the gradual decline in the canopy layer, the WUE under the planting density of D3 exhibited an initial increasing trend and then decreased [24]. This may be due to the stronger light intensity and increased Tr of leaves under the D3 planting density and the more suitable illumination, temperature, and humidity at canopy layer 2, resulting in the maximum WUE. However, with the gradual decline of the canopy layer, the light intensity gradually weakened, leading to a gradual decrease in WUE from canopy layers 3 to 5. Moreover, the WUE under D1 and D2 planting densities exhibited a decreasing trend, possibly due to the light intensity and the vertical distribution of chlorophyll in the canopy layer [39].

CO_2_ is the primary factor for photosynthesis in plants. The content of CO_2_ directly affects the progress of photosynthesis. In photosynthesis, factors such as stomatal state, Tr, and WUE affect the Ci [40]. In this study, under each planting density, with the gradual decline in the canopy layer, the Ci demonstrated a gradual increasing trend. Additionally, with the increase in planting density, the Ci increased gradually. The Ci under the D3 planting density was significantly lower than the other two planting densities. This may be due to the low light intensity and weak light transmittance of leaves at low canopy layers and high planting density. Moreover, the leaves can only use low concentrations of CO_2_, leading to blocked photosynthesis and the accumulation of Ci [5,41].

Leaf phenotypic traits are the result of complex interactions between plant genes and the environment [42]. Leaves, as the main organ of plant photosynthesis and transpiration, are highly sensitive to environmental changes [43,44]. Leaf phenotype exhibits phenotypic plasticity in different environments. Phenotypic plasticity refers to the ability of genotypes or individuals to respond to the environment by changing their phenotypes, which is a key mechanism for plants to adapt to environmental variation [45]. In this study, under the three planting densities, with the gradual decline of the canopy layer, the LA exhibited a gradual increasing trend. Among these, the leaf traits of canopy layer 5 under the D1 planting density were higher compared to the other two planting densities. This may be due to the light protection strategy of plants to increase LA under low-light conditions [46]. Leaves will capture luminous energy by increasing the area, thereby increasing the Pn [47,48,49,50]. Additionally, under the three planting densities in this study, the LL, LW, and LA of canopy layers 3 and 4 under the D2 planting density were higher compared to the other two planting densities. This could be because the light conditions from canopy layers 3 to 4 are more suitable when using the D2 planting density. Excessive light intensity may cause photoinhibition and leaf dormancy [51], while weak light intensity is not conducive to the photosynthesis of plants [52,53]. Optimal illumination under this planting density can regulate the synthesis and distribution of phytohormone by providing energy, promoting poplar growth.

As the main organ of plant photosynthesis, and due to its morphological characteristics and structural composition, leaf phenotype is closely related to photosynthesis and directly affects the efficiency and ability of photosynthesis. Optimizing these characteristics can enhance the efficiency of photosynthesis, leading to improved plant growth and development [54,55]. In this study, correlation analysis revealed that Pn, Tr, WUE, and Gs were positively correlated, while Ci was negatively correlated with other photosynthetic traits. This may be due to the increase in leaf stomatal opening in photosynthesis, leading to intensification of leaf transpiration, accelerated water use, and CO_2_ consumption, resulting in decreased Ci. Furthermore, there were positive correlations among leaf traits, as well as negative correlations between leaf traits and Pn, Tr, WUE, and Gs. Additionally, there were positive correlations between leaf traits and Ci. This may be attributed to the fact that as planting density increases, the canopy closure of the stand correspondingly rises, leading to a significant reduction in light intensity in the lower canopy layer and a consequent decrease in Pn. Under low-light conditions, leaves enhance light capture efficiency by increasing LA to maintain photosynthetic performance. The expansion of leaf area enhances the surface area available for light absorption, effectively offsetting the adverse effects of insufficient light on photosynthesis. This adaptive response not only reflects the plant’s mechanism for responding to environmental changes but also provides critical insights into the impact of stand density on photosynthesis [56].

## 4. Materials and Methods

### 4.1. Study Site

The study site is located in Xinjiang experimental forest farm (47°58′30″–48°05′00″ N, 124°22′30″–124°30′00″ E) of Qiqihar branch of Heilongjiang Academy of Forestry in the Songnen Plain. The average annual temperature is 0.7 °C, with a maximum temperature of 36.5 °C and an annual precipitation of 430 mm. The frost-free period is 120–300 d, and the annual sunshine equals 2715 h. The terrain is mostly flat, with an altitude of about 166–181.6 m. Due to the continental monsoon climate, early spring is characterized by low rainfall and windy conditions, while summer precipitation is concentrated in July and August. Autumn experiences early frost, and winter is cold. The soil types are mainly alluvial sandy soil and chernozem, with a granular soil structure.

### 4.2. Study Design

**Figure 4:**
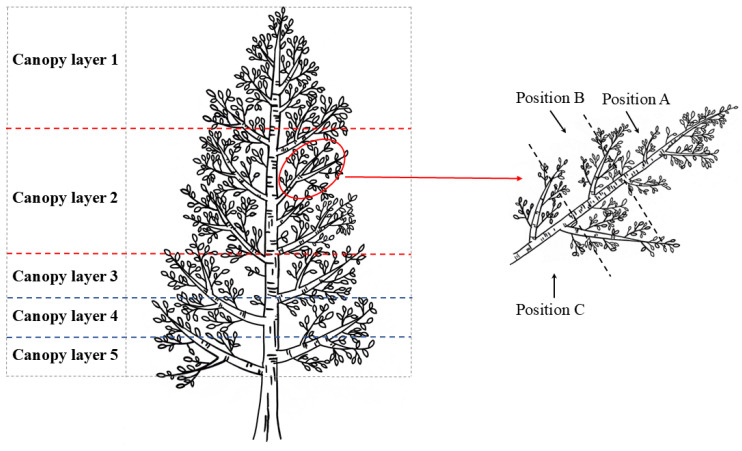
Canopy layer classification diagram. The lateral branch positions from outside to inside are position A, position B, and position C, in turn. Use red lines to divide the canopy of the standard tree into three equal layers from top to bottom, and then use blue lines to further divide the bottom layer into three equal layers.

In July 2023, a photosynthetic test was conducted in the 12-year-density forest of *P. euramericana* ‘N3016’ × *P. ussuriensis* in Xinjiang experimental forest farm. The planting density was 1650 trees·ha^−1^ (D1, 2 m × 3 m × 6 m), 1089 trees·ha^−1^ (D2, 3 m × 3 m × 6 m), and 825 trees·ha^−1^ (D3, 4 m × 3 m × 6 m). The stands were pruned in 2022, with the pruning intensity of no pruning (CK) and tree height 35% pruning (35% pruning). Based on the diameter at breast height data collected one year before and after pruning, the annual diameter at breast height growth under different planting densities was calculated, along with the percentage increase in the diameter at breast height after pruning (Table 3). Without pruning, a tree-by-tree survey was conducted on the diameter at breast height (DBH), tree height, and crown width of the trees within the stand. After calculating the average values, the tree closest to the average values was selected as the standard tree. One standard tree was chosen from each planting density for the experiment. The canopy of the standard tree was divided into five canopy layers from top to bottom (numbered 1, 2, 3, 4, and 5, respectively). Moreover, the first-order lateral branches of each canopy layer were divided into three sites from outside to inside (labeled A, B, and C, respectively). The leaves of each canopy layer were measured for photosynthetic indexes and leaf phenotypes. The poplar canopy layer diagram for classification is displayed in Figure 4.

### 4.3. Measurement of the Photosynthetic Index

**Figure 5:**
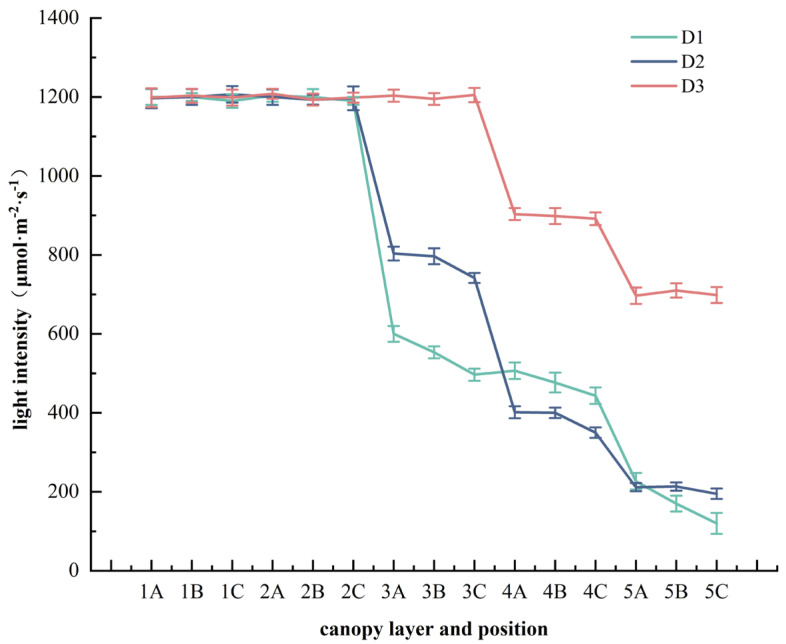
Light intensity of different planting densities at different positions of each canopy layer. The unit of light intensity is μmol·m^−2^·s^−1^. A, B, and C represent the position of the lateral branch, from the outside to the inside, in turn, as position A, position B, and position C; the abscissa 1, 2, 3, 4 and 5 represent canopy layer 1, canopy layer 2, canopy layer 3, canopy layer 4 and canopy layer 5 respectively; D1 approximately 1650 trees·ha^−1^, D2 approximately 1089 trees·ha^−1^, D3 approximately 825 trees·ha^−1^.

During the measurement of the photosynthetic index, branches in the same direction of each canopy layer were selected for measurement. At each position, three mature leaves were selected for measurement, with each leaf measured three times. The average value was taken as the experimental analysis data. The measurements took place from 9:00–11:30 a.m. on sunny days. Before the measurement, an elevator was used to reach the different canopy layers of the standard wood, while the light intensity was measured in different canopy layers and lateral branches of standard wood of the photosynthesis system by a quantum sensor (LI-6400, LI-COR, Inc., Lincoln, NE, USA) (Figure 5). The light intensity of the photosynthesis system was set according to the measured data, and the CO_2_ concentration was set at 400 μmol/mol. Subsequently, the photosynthetic indexes of each canopy layer were measured, including Pn, Tr, Ci, and Gs. WUE was calculated by the ratio of Pn to Tr.

### 4.4. Measurement of Leaf Phenotype

After measuring the photosynthetic index and leaf fresh weight, leaves from different positions within each canopy layer were photographed, and their leaf area (LA), leaf length (LL), and leaf width (LW) were determined using ImageJ software (version 1.52j; NIH., Bethesda, MD, USA). Finally, the leaves were placed in an oven at 105 °C for 30 min and then dried at 80 °C until a constant weight was achieved to determine the leaf dry weight. The leaf water content (LWC) was calculated using the leaf fresh weight and dry weight [57].

(1) $$leaf\;water\;content\left(LWC\right)=\frac{fresh\;weight-dry\;weight}{fresh\;weight}\times 100\%$$

### 4.5. Data Analysis

The data were analyzed using EXCEL and Statistical Package for the Social Sciences software (version 26.0; IBM Corp., Armonk, NY, USA) and plotted using Origin 2024 software. Analysis of variance (ANOVA) was used to analyze the differences in photosynthetic and leaf traits in different canopy layer positions of different planting densities.

The ANOVA linear model is given below [58]:

(2) $$X_{ijkl}=\mu +D_{i}+C_{j}+P_{K}+{DC}_{ij}+{DP}_{ik}+{CP}_{jk}+{DCP}_{ijk}+e_{ijkl}$$

where *μ* is the overall mean, *D_i_* is the density effect, *C_i_* is the canopy layer effect, *P_k_* is the position effect, *DC_ij_* is the interaction effect of density and canopy layer, *CP_ij_* is the interaction effect of canopy layer and position, *DCP_ijk_* is the interaction effect of density and canopy layer and position, and eijkl is the random residual.

The phenotypic correlation coefficient (*r_p_*_12_) was calculated using the following formula [59]:

(3) $$r_{p12}=\frac{{Cov}_{p12}}{\sqrt{\sigma_{p1}^{2}.\sigma_{p2}^{2}}}$$

where *Cov_p_*_12_ denotes the phenotypic covariance between characters 1 and 2 and $\sigma_{p1}^{2}$ and $\sigma_{p2}^{2}$ denote the phenotypic variance of characters 1 and 2, respectively.

## 5. Conclusions

Determining the effective canopy layer height of trees under reasonable planting density can better promote the growth of trees. In this study, the photosynthetic traits and leaf traits of different canopy layer positions of poplars under different planting densities were analyzed. The results revealed that different planting densities significantly affected the photosynthetic and leaf traits at different positions of each canopy layer. With the decrease in planting density, the photosynthetic capacity of poplar gradually increased. Under the planting density of 825 trees·ha^−1^, different canopy layers could carry out effective photosynthesis, and the living branches of each canopy layer were found to be effective for photosynthesis. Effective photosynthesis can occur in canopy layers 1 to 4 under planting densities of 1650 and 1089 trees·ha^−1^, while canopy layer 5 has relatively weak photosynthetic capacity. As planting density changes, the lateral branches move from outside to inside, and the LA gradually increases. The poplar captures more luminous energy in low light intensity by increasing the LA to promote photosynthesis. This study can provide new insights into understanding the photosynthetic efficiency and growth strategies of poplar under different planting densities, while also offering theoretical support for optimizing stand management and enhancing productivity.

## Figures and Tables

**Table 1:** Analysis of variance table showing different planting densities, canopy layers, and positions of photosynthetic traits and leaf traits.

Categories	Traits	F-Value						
		Density	Canopy	Position	Density × Canopy	Density × Position	Canopy × Position	Density × Canopy × Position
Physiological traits	Pn	315.676 **	1639.756 **	922.406 **	84.363 **	28.456 **	92.659 **	24.717 **
	Tr	135.387 **	727.99 **	242.421 **	26.529 **	16.087 **	20.247 **	10.278 **
	WUE	545.108 **	197.844 **	111.491 **	49.769 **	21.673 **	23.073 **	15.601 **
	Gs	347.909 **	341.136 **	349.697 **	34.457 **	25.896 **	41.385 **	16.992 **
	Ci	579.216 **	192.267 **	21.605 **	24.267 **	3.759 **	5.657 **	13.169 **
Morphological traits	LL	18.961 **	14.980 **	2.417 ns	18.612 **	1.273 ns	6.535 **	1.847 *
	LW	63.791 **	59.696 **	5.272 **	12.613 **	2.577 *	5.587 **	2.124 **
	LA	83.041 **	62.832 **	8.020 **	15.960 **	2.150 ns	6.322 **	3.641 **

** Significant at the 0.01 level; * significant at the 0.05 level; ns, not significant; Pn, instantaneous photosynthetic rate (μmol·m^−2^·s^−1^); Tr, transpiration rate (mmol·m^−2^·s^−1^); WUE, water use efficiency (μmol·m^−2^·s^−1^); Gs, stomatal conductance (mol·m^−2^·s^−1^); Ci, intercellular CO_2_ concentration (μmol·mol^−1^); LL, leaf length (cm); LW, leaf width (cm); LA, leaf area (cm^2^).

**Table 2:** Correlation analysis between photosynthetic traits and leaf traits.

Traits	Pn	Tr	WUE	Gs	Ci	LL	LW	LA
**Tr**	0.717 **							
**WUE**	0.784 **	0.154 **						
**Gs**	0.586 **	0.906 **	0.025					
**Ci**	−0.705 **	−0.189 **	−0.868 **	0.010				
**LL**	−0.155 **	−0.099 *	−0.171 **	−0.043	0.195 **			
**LW**	−0.341 **	−0.284 **	−0.259 **	−0.201 **	0.268 **	0.671 **		
**LA**	−0.529 **	−0.345 **	−0.532 **	−0.209 **	0.545 **	0.400 **	0.471 **	
**LWC**	−0.534 **	−0.586 **	−0.305 *	−0.422 **	0.266	0.236	0.444 **	0.368 *

* Significant at the 0.05 level; ** significant at the 0.01 level. Pn, instantaneous photosynthetic rate (μmol·m^−2^·s^−1^); Tr, transpiration rate (mmol·m^−2^·s^−1^); WUE, water use efficiency (μmol·m^−2^·s^−1^); Gs, stomatal conductance (mol·m^−2^·s^−1^); Ci, intercellular CO_2_ concentration (μmol·mol^−1^); LL, leaf length (cm); LW, leaf width (cm); LA, leaf area (cm^2^); LWC, leaf water content (%).

**Table 3:** The mean value and percentage of DBH growth in one year under different treatments.

Planting Density	DBH1 (CK)	DBH1 (35% Pruning)	Percentage of DBH Growth
D1	0.65	0.72	10.77%
D2	0.75	0.84	12.00%
D3	0.90	0.85	−5.56%

The unit of 1-year increase in diameter at breast height (DBH) is cm. CK indicates no pruning treatment, and 35% pruning represents a pruning intensity of 35% of the tree height. D1, approximately 1650 trees·ha^−1^; D2, approximately 1089 trees·ha^−1^; D3, approximately 825 trees·ha^−1^.

## Data Availability

The datasets analyzed during the current study are available from the corresponding author on reasonable request.
